# Biocompatible and Biodegradable Functional Polysaccharides for Flexible Humidity Sensors

**DOI:** 10.34133/2020/8716847

**Published:** 2020-04-09

**Authors:** Lili Wang, Zheng Lou, Kang Wang, Shufang Zhao, Pengchao Yu, Wei Wei, Dongyi Wang, Wei Han, Kai Jiang, Guozhen Shen

**Affiliations:** ^1^State Key Laboratory on Integrated Optoelectronics, College of Electronic Science and Engineering, Jilin University, Changchun 130012, China; ^2^State Key Laboratory for Superlattices and Microstructures, Institute of Semiconductors, Chinese Academy of Sciences, Beijing 100083, China; ^3^Sino-Russian International Joint Laboratory for Clean Energy and Energy Conversion Technology, College of Physics, Jilin University, Changchun 130012, China; ^4^Laboratory of Theoretical and Computational Chemistry, Institute of Theoretical Chemistry, Jilin University, Changchun 130012, China; ^5^International Center of Future Science, Jilin University, Changchun 130012, China; ^6^Institute & Hospital of Hepatobiliary Surgery, Key Laboratory of Digital Hepatobiliary Surgery of Chinese PLA, Chinese PLA Medical School, Chinese PLA General Hospital, Beijing 100853, China

## Abstract

Using wearable devices to monitor respiration rate is essential for reducing the risk of death or permanent injury in patients. Improving the performance and safety of these devices and reducing their environmental footprint could advance the currently used health monitoring technologies. Here, we report high-performance, flexible bioprotonic devices made entirely of biodegradable biomaterials. This smart sensor satisfies all the requirements for monitoring human breathing states, including noncontact characteristic and the ability to discriminate humidity stimuli with ultrahigh sensitivity, rapid response time, and excellent cycling stability. In addition, the device can completely decompose after its service life, which reduces the risk to the human body. The cytotoxicity test demonstrates that the device shows good biocompatibility based on the viability of human skin fibroblast-HSAS1 cells and human umbilical vein endothelial (HUVECs), illustrating the safety of the sensor upon integration with the human skin.

## 1. Introduction

Electronic sensors, such as wearable physiological monitoring and stimulating devices, have been widely used in biomedical applications [1, 2]. Such devices need to be flexible to accommodate strains from repeated movement while maintaining secure contact with the human skin, resulting in minimal injury from long-term exposure and offering maximum comfort to the wearer [3]. Although previous biocompatible wearable systems for health monitoring have demonstrated good long-term stability during the physiological monitoring [4], obtaining suitable materials that fulfill all these requirements simultaneously is challenging due to the trade-offs between biocompatibility and high performance. At present, the most common methods to improve the compatibility between devices and the human body focus on the design of the device structure [5–7]. For example, ultrathin electronic devices with mechanical properties similar to biological systems will offer improved mechanical matching across the device-tissue interface to reduce the adverse immune response from the human-computer interaction [8]. In addition, the development of low-cost, intrinsically biocompatible and biodegradable materials that provide excellent mechanical properties without compromising their sensing performance is another approach to fabricate high-performance flexible sensors [2, 9].

**Figure 1:**
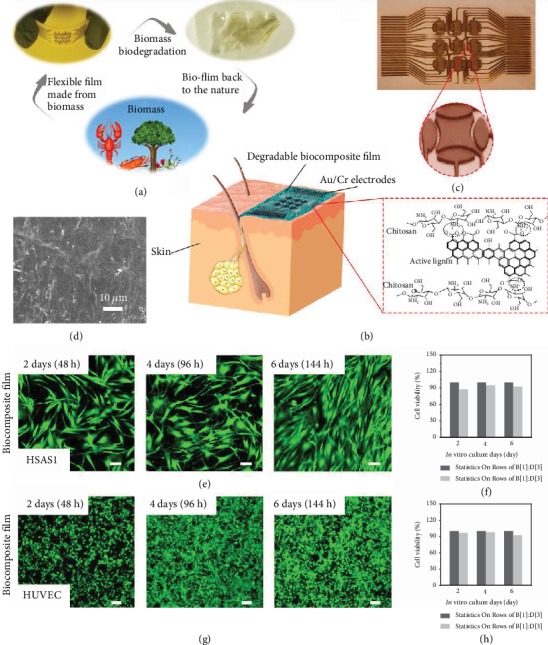
A biocompatible and biodegradable bioprotonic flexible sensor. (a) Schematics of a likely life cycle of a degradable natural functionalize polysaccharide film. First, functionalized polysaccharide which came from nature is made into biofilm. The biofilm can be degraded via biodegradation and sent back to nature to reduce the environmental footprint. (b) Schematics of a biodegradable and biocompatible humidity sensor attached on the skin. The inset shows the chemical structure of natural functionalized polysaccharide film used to fabricate the sensor. (c) Scanning electron microscope (SEM) image of natural functionalized polysaccharide film. (d) Optical image of the electronic structure of a flexible device. The inset shows the local enlarged image of a device. (e, g) Confocal laser scanning microscopy images of stained human skin fibroblast-HSAS1 and HUVEC that were cultured on a functionalized polysaccharide film. Scale bar: 100 *μ*m. (f, h) Cell viability of human skin fibroblast-HSAS1 and HUVEC on 2, 4, and 6 d of in vitro culture (*n* = 3 measurements).

Natural materials, those produced from renewable and abundant feedstocks and having intrinsically biocompatibility and biodegradability, have been used in various applications [1, 10], such as neural implants [8], batteries [11], bioengineering [12], printed electronics [13–15], and drug delivery [16]. In this work, we report flexible moisture-triggered, bioprotonic devices made entirely of biocompatible and biodegradable natural polysaccharide materials. Polysaccharide-based flexible bioprotonic devices can be synthesized from natural materials and decomposed back to the environment (Figure 1(a)). We believe that this sensor satisfies all the requirements for monitoring human breathing states, including biodegradability, biocompatibility, the ability to discriminate humidity stimuli with excellent sensitivity (2084.7%), and fast response times (29 ms). In addition, our sensors have been successfully applied to both a smart, noncontact multistage switch and a novel, flexible noncontact screen for smart devices. The cytotoxicity test confirmed that the sensor exhibits excellent biocompatibility and biodegradability, eliminating the injury associated with the integration of sensors with the human skin, demonstrating the potential of these sensors in future human-machine interaction systems.

## 2. Results

### 2.1. Device Architecture

Here, we present a biodegradable and biocompatible humidity sensor that attaches to the skin to monitor human breathing and ambient humidity (Figure 1(b)). The device was prepared by depositing ~60/10 nm thick Au/Cr electrodes on top of an ~20 *μ*m thick degradable biocomposite film (Figure S1), and the corresponding tilted and magnified optical images are shown in Figure 1(c) and its inset, respectively. The assembly of the sensor is simple and involves spin coating and thermal evaporation (Figure S2) [17]. Functionalized polysaccharides can be spin-coated onto flexible substrates or directly deposited onto the surface of the human skin, which further improves the flexibility of the device and makes the sensor easier to apply to the skin surface [18]. This simple process is highly scalable. The degradable biocomposite film is made of biocompatible functionalized chitosan with a relatively smooth planar structure (Figure 1(d)). Because natural biomaterials have ideal degradation kinetics, are easily processed and suitable for large-scale production, and offer excellent biocompatibility upon degradation, they can be rapidly translated in the clinic for health monitoring [1, 19]. In contrast, the cytotoxicity of synthetic materials may limit the applicability of most biodegradable composite materials in the field of biomedicine, because the degradation of synthetic materials may release toxic substances that endanger human health and cause environmental pollution. The key component of our device is a natural, functionalized polysaccharide, a chitin derivative, which not only is biodegradable and biocompatible but also has unique bioprotonic properties [10, 20].

### 2.2. Cell Biocompatibility In Vitro

First, the animal-derived polysaccharide (chitosan) has been subject to extensive biocompatibility studies that have demonstrated that it offers cell and tissue biocompatibilities comparable to those of other reference biodegradable polymers for transient applications. In the present study, we further confirmed the biocompatibility of the natural degradable biocomposite film for direct interfacing with the human skin or living tissue via cytotoxicity tests. In the biocompatibility tests, human skin fibroblast-HSAS1 and HUVEC were exposed to the degradable biocomposite film (~20 *μ*m) (Figures 1(e) and 1(g)). Compared with the control group (standard cell culture dish, see Figure S3), the confocal laser scanning microscopy images showed that the degradable biocomposite film had no obvious effect on the cell viability of the human skin fibroblast-HSAS1 and HUVECs (Figures 1(f) and 1(h)). Namely, the survival rates of human skin fibroblast-HSAS1 and HUVECs were 92.3 ± 2.7% and 92.4 ± 3.1%, respectively, after 6 d (144 h). These results indicate that the natural degradable biocomposite film is biocompatible and safe for use on the skin [21].

### 2.3. Protonic Conductive Measurement

**Figure 2:**
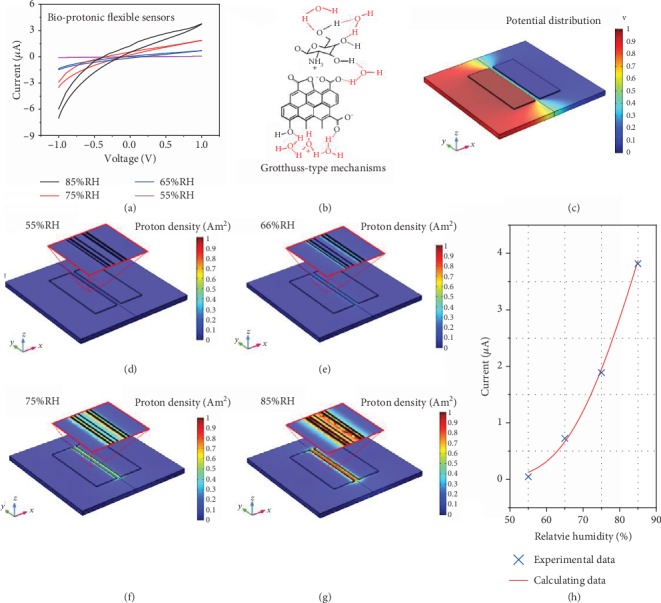
Simulated proton density in degradable biocomposite film-based flexible device. (a) Current-voltage (I-V) curves of degradable biocomposite film-based flexible device measured under different humidity conditions. (b) Schematic diagram of proton transition along a chain of hydrogen-bonded water molecules, as postulated to occur for the Grotthuss mechanism. (c) Electric potential and (d–g) proton density distribution in degradable biocomposite film-based flexible device under different humidity conditions. (h) Current change according to FEM modeling for degradable biocomposite film-based flexible humidity sensors compared with the experiment results (55-85%RH).

In addition to their established biocompatibility upon degradation, excellent sensing performance is also an important consideration for this application.  Figure 2(a) shows representative I-V curves of the device under different relative humidity (RH) conditions. We observed increases in current as different humidity sources were moved closer to the humidity sensor. During the experiment, the hysteresis increases with increasing humidity, which may be due to the increase in charge accumulation/depletion [20]. The increase in current in degradable biocomposite film under humid conditions should be due to the formation of hydrogen bond networks between the water and polysaccharides in the polysaccharide films [22]. Higher water absorption results in a more proton-conducting hydrogen bonds, which form a three-dimensional network, and protons can move along these chains via Grotthuss-type mechanisms [20, 23]. The phenomenon of proton conduction is ubiquitous in biomacromolecule-derived materials. For degradable biocomposite film, we propose that the carboxyl group on the chitosan side chain is deprotonated, donating a proton to the surrounding proton wires and forming a protonic conductor with H^+^ as the major charge carrier (Figure 2(b)) [22]. Namely, the proton current in the degradable biocomposite film at 75%RH is threefold higher than that at 65%RH. At 85%RH, more water was adsorbed on the surface of the degradable biocomposite film, forming more hydrogen-bonded proton wires, which increased proton transfer, thus increasing the current [24]. To rationalize the experimental results and estimate the effects of humidity on the proton density in the channels of the degradable biocomposite film, we simulated changes in the proton charge density in degradable biocomposite film using the finite element method (FEM) (detailed parameters are provided in Materials and Methods). The mechanistic model is based on the Gompertz equation, and when the electronic density is replaced by the proton density (see Materials and Methods), the change in the proton charge density at different humidities was obtained. The thickness of the degradable biocomposite film was 20 *μ*m (Figure S1). The modeling results showed (Figure 2(c)) no obvious changes in the potential distribution at the same humidity due to the homogeneity of the degradable biocomposite film; that is, the electronic field distribution is not affected by humidity. Figures 2(d)–2(g) show that exposing the flexible device to a humid environment leads to a change in proton density. The proton density increases with increasing humidity (Figure S4). In a high-humidity environment (85%RH), a high number of protons gather in the conductive channels to form a highly conductive region (Figure 2(g)). Similar effects of humidity on current and charge density distributions have been reported for other organic humidity sensors [20, 25].  Figure 2(h) shows the simulated and experimental current responses of the degradable biocomposite film-based flexible humidity sensor at 55%RH, 65%RH, 75%RH, and 85%RH. The results of the simulations were close to the experimental data at high humidity and show a linear dependence of current on humidity at 55-85%RH, which demonstrated that the degradable biocomposite film-based flexible humidity sensor responds to humidity [20].

### 2.4. Humidity-Sensing Measurements

**Figure 3:**
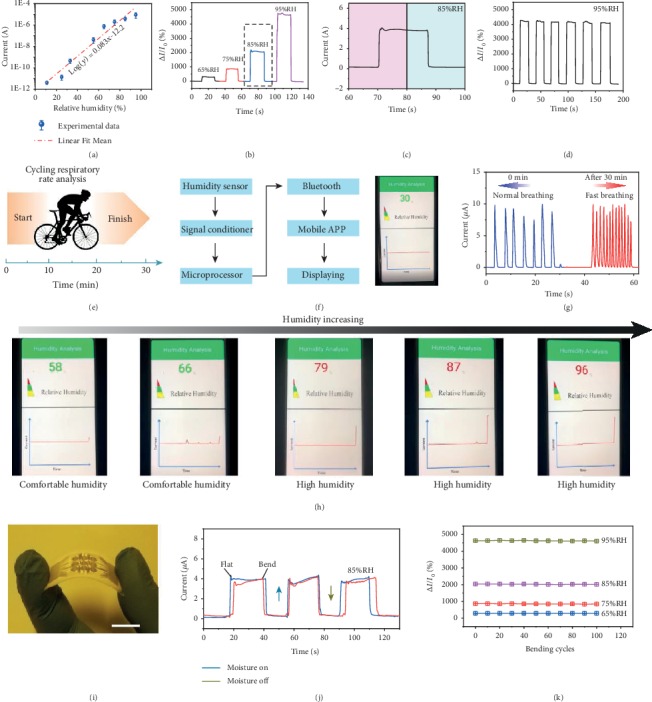
Humidity-sensing properties. (a) Current-voltage characteristics under different RH with an applied bias voltage fixed at 1 V (*n* = 3 measurements). (b) Sensitivity plots of flexible sensors under 65%RH-95%RH conditions. (c) Dynamic current change (1 cycle) of flexible sensors at 85%RH. (d) The humidity switching characteristic of the device at 95%RH. (e) Schematic diagram of humidity monitoring through exercise-induced respiration rate change. (f) Illustration of the sensing platform for real-time detecting, signal processing, and wireless transmission. (g) Measured respiration rate changes during the exercise experiment. (h) Real-time monitoring of humidity in mobile phone software from low humidity to high humidity. (i) Optical image of flexible humidity sensors. Scale bar: 1 cm. (j) Dynamic sensing curves of the flexible sensor under flat and bend state for 85%RH. (k) Long-term stability of the flexible sensor with 65%, 75%, 85%, and 95%RHs under 120 bending cycles.

Finite element analysis (FEA), which combines both humidity and proton density simulations, revealed that the sensor has high humidity-sensing performance. The increase in the sensitivity is due to the increase in the number of water molecules adsorbed by the biomaterial in the channels at the same relative humidity, which results in more proton density for transport and substantially influences the conductivity of the channel.  Figure 3(a) shows the current change in bioprotonic flexible sensors under different RH levels at a bias of 1 V. The current response of bioprotonic flexible sensors exhibits a linear detection range from 11% to 95%RH that allows accurate conversion and prediction of the relationship between RH and the current. In addition, the current in the bioprotonic flexible sensors changes by a factor of more than 106, indicating that these bioprotonic flexible sensors have excellent humidity responses under both high- and low-humidity conditions. The dynamic sensitivity (defined as the relative current changes [17], *S* = Δ*I*/*I*_0_, where Δ*I* = *I*‐*I*_0_, *I* represents the current with a moisture load, and *I*_0_ represents the current without a moisture load) curves (Figure 3(b)) exhibit linear increases in sensitivity over a wide dynamic range (RH values ranging from 65% to 95%) (Figure S5a). The response/recovery times (*τ*_res_/*τ*_recov_), defined as the time required for 90% of the current increase or decrease [16], were evaluated to be approximately 29 and 30 ms (Figure 3(c)), which are faster than the values reported for other humidity sensors (Figure S5b and Table S1). The humidity switch characteristic reveals the excellent stability and reversibility of our sensor (Figure 3(d)). The sensitivity can reach approximately 4241.7% at 95%RH, and the flexible sensors still have an ultrafast response and recovery time even after 6 cycles (Figure S6). Together, these results confirm that our sensor can be used as a humidity signal detector and provide fast real-time responses.

To evaluate the ability of these sensors to detect physiological behaviors related to humidity in the human body, we measured the respiration rate change before and after human exercise [26]. Since respiratory rate is a key vital sign, accurate detection of changes in respiratory rate can effectively reduce the risk of death and permanent injury [27].  Figure 3(e) illustrates a time-course panel of the changes in the respiratory rate with exercise. As a wireless, wearable real-time monitoring system, as shown in Figure 3(f) and Figure S7, the completely integrated humidity sensor includes a signal conditioner, microprocessor, Bluetooth, mobile app, and real-time display to transmit electronic signals to a mobile phone and achieve in situ personalized humidity monitoring. The ultrafast performance of flexible sensors allows fine features associated with moisture modulation in the respiration rate to be captured during human exercise. As shown in Figure 3(g), the sensor could efficiently follow normal breathing (∼4.2 s) and relatively fast breathing (∼1.5 s), suggesting athletes (Figure S8) [28]. Patients or others may use this humidity sensor to monitor routine RH changes and respiratory status to track health-related changes. In addition, through our wireless system, people can read the RH of the surrounding environment in real time and then select appropriate treatments to improve comfort. Our integrated systems can rapidly display the ambient RH and send a reminder based on a designated RH threshold. Examples of the real-time display of humidity via mobile phone software under comfortable humidity and high-humidity conditions are displayed in Figure 3(h). In addition, humidity-sensing tests using the flexible sensors (Figure 3(i) were conducted with the sensors flattened and bent (angle: 60°C) (Figure 3(j)) at different angles (Figure S9), and similar sensing behaviors were observed. The sensitivity of our sensors was maintained at different RH levels throughout more than 120 bending cycles (Figure 3(k)), demonstrating good durability and stability. These results offer broad development prospects for applications involving wearable medical devices.

### 2.5. Smart Noncontact Sensor

**Figure 4:**
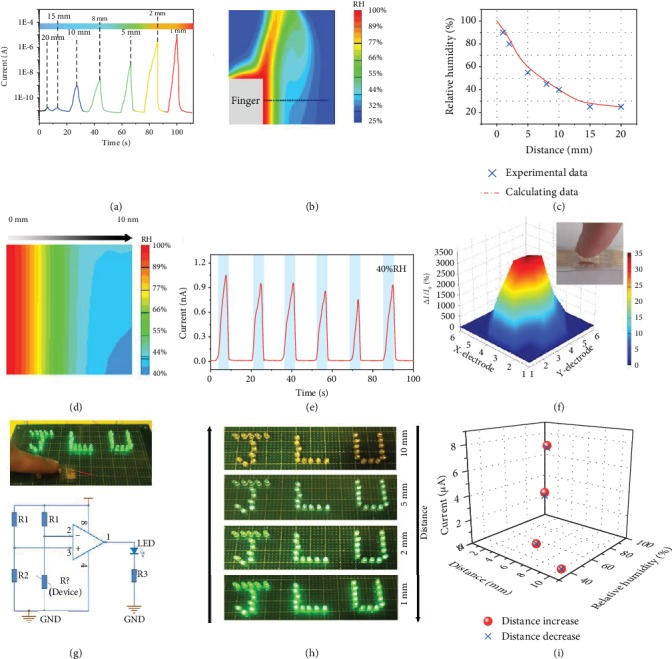
Smart noncontact sensor. (a) Dynamic humidity-sensing curves of flexible sensor with different finger distances. (b) Cross-sectional FEM image shows humidity distribution with lateral color gradient around the finger position (20 mm). (c) Relationship curves of RH and distance (x) conducted by FEM analysis, which is close to the estimated value from the sensor. (d) Enlarged FEM image of humidity distribution around the finger position (10 mm). (e) Repeated response circles of the noncontact sensor (10 mm). (f) The 3D mapping of the noncontact sensor when the fingertip approaches the relative center area of the device. The inset is the photograph of this process. (g) Demonstration (top) and circuit diagram (bottom) of the noncontact smart switch system for two LED lights. (h) Optical image of reversible LED light brightness change of the noncontact sensor under different distances or RHs. (i) Reversible current response of the noncontact sensor under different distances and RH.

In addition to its application in medicine, our sensor can also be used as a switch in contactless controllers due to its superior response to fingertip humidity [29, 30]. Positioning the surface of the composite film at a vertical distance of 1 mm from one side of the finger surface leads to a current change along a distance between 1 and 20 mm from the fingertip, as shown in Figure 4(a). The conductance of the degradable biocomposite film offers fast, reproducible, and selective response to changes in RH, which is a key performance index for position-sensitive user-controlled interfaces. Because the humidity of the environment surrounding the fingertip is distance dependent, the above sensing scheme provides an interesting platform for the development of different noncontact, interactive directional interfaces (i.e., lateral- and height-sensitive interfaces) [31]. FEA simulates the relationship between the output humidity distribution and the distance of one finger. In Figures 4(b) and 4(d), the moisture distribution of the environment surrounding a finger is illustrated in values of RH [32]. The average RH of the air surrounding the whole finger is 44%RH (Supplementary Figure 10). The relative humidity of the finger surface is approximately 95%, but the RH of the finger at a distance of 20 mm is approximately 25% (Figure 4(b)). Therefore, humidity varies as a function of distance. The difference in humidity from the finger surface to a distance of 20 cm is 70%RH. Namely, as the distance from the finger increases, the RH gradually decreases (Figure 4(b)). This is very consistent with the humidity-sensing performance, as in the distance range from 20 mm to 1 mm, the sensing signal rapidly increases (Figure 4(a)) because the humidity gradient around the finger varies with distance. The relationship between RH and distance from the fingertip was identified via FEA, and the results of the simulation are close to the experimental data (Figure 4(c)). From the structural colors of the gradient, the color at an actual distance of 1.0 mm corresponds to the sensing signal 90-95%RH. The RH corresponding to the structural color at a distance of 10 mm corresponds to approximately 40%RH (see Figure 4(d)). Humidity switch characteristics were also achieved by repeatedly approaching the device, revealing the excellent stability and reversibility of our device (Figure 4(e) and Figure S11).

To further indicate the noncontact humidity-sensing characteristics of this sensor array, one fingertip held close to the sensor matrix allows the individual current of each pixel to be measured. The pixel currents in the sensor array rapidly increased when the finger was near the center of the sensor array, as illustrated in Figure 4(f). As a result, the noncontact devices have high, pixel-level resolution. Furthermore, we assembled a smart noncontact control switch system that utilized the sensitive response of the sensor to fingertip humidity at different distances (Figure 4(g), top image). As the fingertip approaches and leaves the device, a light-emitting diode (LED) will turn on and off in sequence. An amplifying circuit was prepared to implement this function (Figure 4(g), bottom image). When a finger approached the surface of the degradable biocomposite film-based flexible sensor, the current in the sensor increased, and the LED lights up immediately. That is, the switching state of the LED can be used to signal a change in humidity. When the finger moves away, the LED turns off quickly because the hydrolysis triggers a decrease in current (Figure S12). As expected, the brightness of the LED can be controlled by the distance from the finger to the device. Figure 4(h) illustrates a sequence of optical images generated by exposing this device with J-, L-, and U-shaped LEDs to different humidity conditions and cyclically controlling the distance between the finger and the sensor. When the distance between the finger and the device is relatively large (10 mm), the LED emitted relatively weak yellow light. Interestingly, as the finger gradually approached the device (from 5 mm to 1 mm), the emission of the LED changed from light green to bright green, which is in good agreement with the change in the current. Subsequently, the change in the periodic brightness of the LED was reversed as the finger moved away from the device. When the distance between the finger and device increases gradually from 1 mm to 10 mm following the previous step, the emission of the LED returns from bright green to weak yellow, and the emission of the LED is completely reversible. Similar reversible current variations with humidity or distance are shown in the figure, further illustrating that our devices can be used as noncontact control devices (Figure 4(i)).

### 2.6. Biodegradation Tests

**Figure 5:**
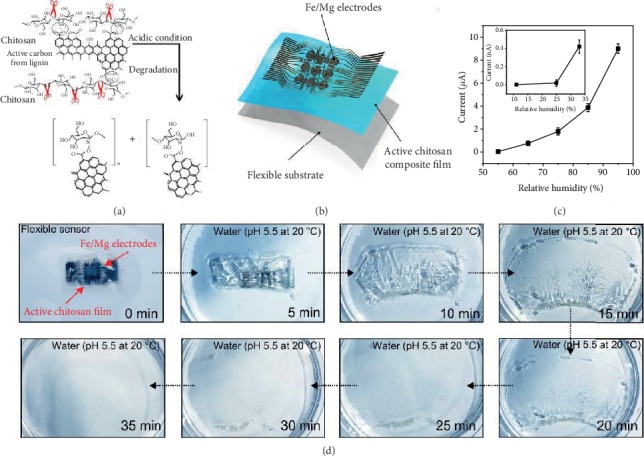
Biodegradation tests of bioprotonic flexible devices. (a) The mechanism for acid-catalyzed hydrolysis. (b) Illustration of biodegradable bioprotonic flexible devices with a biodegradation materials and electrode. (c) Humidity characteristic of flexible device using Fe/Mg as electrodes (*n* = 3 measurements). (d) Optical images of degradation of bioprotonic flexible devices upon insertion into an aqueous solution (pH 5.5) at room temperature.

Assessing the biodegradability of flexible sensors is crucial not only for modern biomedical technologies but also for cutting-edge wearable electronics [33]. The unique feature of our sensors is their ability to dissolve completely under acidic conditions after use, resulting in sensors with a smaller environmental footprint of sensors and facilitating advanced health monitoring and treatment technologies [19]. Acid-driven degradation plays a major role in natural polymer-based structures. Functionalized chitosan is a biodegradable polysaccharide composed of esters, and it can be decomposed into oligomers by acid hydrolysis. The monomers can be further decomposed by the immune systems of microorganisms or organisms in the environment. This process is enough to reduce the demand for invasive and expensive retrieval procedures. So far, gold is used as the electrode for most organic disintegrable electronics [34, 35]. Although gold is biocompatible and has been widely used in implantable and wearable electronic devices, it is not dissolvable and cannot “physically disintegrate” [36, 37]. To achieve completely disintegrable “transient” electronics, a biodegradable metal system, Mg/Fe, was chosen as the electrode for the natural biocomposite film because of its easy processing, biocompatibility, and rapid rate of hydrolysis (Figure 5(a)) [21]. The multilayered design and the corresponding fabrication process are presented in Figure 5(b) and Figure S2, respectively. Figure 5(c) shows the humidity-sensing performances of bioprotonic flexible sensors based on Mg/Fe electrodes. Compared with gold electrodes (Figure S13), devices based on Mg/Fe electrodes exhibit similar humidity-sensing performances. In addition to their high sensitivity, a key challenge for these bioprotonic flexible devices is their biodegradability. Immersion in an aqueous solution illustrates the process leading to complete dissolution into biocompatible end products. The degradation characteristics of a free-standing bioprotonic flexible device placed into a transparent glass petri dish in an aqueous solution at pH 5.5 at room temperature are shown by a series of optical images taken at different time points (Figure 5(d)). The biocomposite film dissolves by a relatively uniform hydrolysis process. In the first 10 min, the transparency of the biocomposite film decreased significantly, which is consistent with the initial stages of swelling, water absorption, and hydrolysis. In this case, the Fe/Mg electrodes degrade quickly, usually within 25 min. The device degrades completely within 35 min, and the onboard electronics are destroyed. In addition, toxicity studies showed the good biocompatibility of the biocomposite film after 6 d, with no statistically significant difference between exposure to the biocomposite film and the control sample (Figure S14). Therefore, our devices will not cause any harm to the human body or biological environments.

## 3. Discussion

The bioprotonic flexible devices based on the functional polysaccharides reported here show high humidity sensitivity for monitoring the human body and the environment, and the devices are fully biocompatible and biodegradable, allowing the safe monitoring of patients while avoiding the risks of permanent injury associated with the integration of devices with the human skin and reducing the environmental footprint of these devices. These bioprotonic flexible devices exhibit high-humidity sensitivity, great flexibility, and excellent noncontact characteristics. These features will be useful for monitoring ambient conditions and various diseases of the human body, from human environmental comfort to chronic diseases. In addition, utilizing the rapid response of the sensor to fingertip humidity at different distances, a smart noncontact control switch system was successfully prepared. Thus, this is suitable for practical flexible sensors for use in stimulating, recording, sensing, and medical monitoring and treatment, not only in the medical applications explored here but also in the field of intelligent human-machine systems.

## 4. Materials and Methods

### 4.1. Functionalized Polysaccharide Solution Preparation

In brief, 0.9 g of chitosan and 0.1 of active lignin (0.1 g) were added to 0.5 M, 10 mL of acetic acid. An advanced ultrasound instrument (Cole Parmer 750 W) was used to treat the mixture with 30% power for 30 min. The resulting dispersion to form homogeneous solution was stirred for 20 h, and the supernatant was retained to be utilized for the biocomposite film forming.

### 4.2. Device Fabrication

The sensor was assembled as described in Figure S9. A chitosan and active lignin mixed solution (9 : 1) was spin-coated for 45 s at 500 r.p.m. and then at 1,500 r.p.m. for 1 min to yield a film with a thickness of approximately 20 *μ*m on a flexible PI substrate as the sensing layer. The flexible PI substrate was ultrasonicated in acetone, deionized water, and isopropanol and then treated with oxygen plasma for 1 min at 150 W before spin coating (Technics MicroRIE Series 800). To the process for fabricating the bioprotonic flexible humidity sensors on a biocomposite film was the same as that previously reported by our group [38, 39]. Au/Cr (60/10 nm thick) electrode patterns were thermally evaporated from a 20 *μ*m thick biocomposite film via a shadow mask. The first layer of Cr is used to generate nucleation sites on the 60 nm gold electrode layer deposited at 150°C, which was used for adhesion.

### 4.3. Fabrication of Biodegradable Metal Electrodes

The electrodes were fabricated by evaporating Mg/Fe (60/10 nm thick) on top of a 20 *μ*m thick functionalized polysaccharides biocomposite film via spin coating and thermal evaporation.

### 4.4. Characterization of the Sensor

The humidity measurement set-up consisted of a Keithley 4200-SCS System SourceMeter (Keithley) used in combination with a humidity generator (DHD-II), while the RH was measured with a humidity generator (DHD-II). Measurements were performed in a different controlled RH atmosphere at room temperature. Digital cameras (Canon, Japan) are used to collect optical images of flexible devices.

### 4.5. Simulations

#### 4.5.1. Electrical Property Simulations

Electrical (in this case, proton) properties of the materials were obtained by using the Gompertz equation together throughout a finite element method (COMSOL, 5.3a). The relationship between conductivity of the materials and RH was determined by solving the following equation:

(1) $$\begin{array}{c} G=a∙e^{-e^{-k∙\left(H_{d}-H_{d0}\right)}}, \end{array}$$

where *G* represents the conductivity of the biocomposite film, *a* represents the limit saturation value (when humidity reaches infinity), and *H*_*d*_ represents RH. Electrical (in this case, proton) properties of our materials were affected by exposure to different RH levels. To calculate the conductivity of the biocomposite film, we first estimated *a* = 1.29707*e*^−6^, *H*_*d*0_ = 84.74426, and *k* = 0.04437 in this case. The electrical properties of our materials were then calculated by imported COMSOL tool.

#### 4.5.2. RH Distribution Simulations

RH distributions in the surrounding finger were simulated by using a CFD model, Fluent (version 6.1). In this model, air acts as a mixture of water vapor and dry air, and the finger acts as fixed fluids with ordinary wall characteristics. This makes it possible to simulate the transfer of moisture on the surface of the finger. Here, we choose a finger with a thickness of 10 mm and used one side of the finger as a fixed fluid wall. The humidity distribution was simulated in the range of 30 mm × 30 mm on one side. Relative pressure in the set area is 0, finger temperature is 37°C, and finger surface humidity is 95-100%RH. The RH distribution was estimated by the simple equation of Navier-Stokes.

### 4.6. Cell Biocompatibility In Vitro

Human skin fibroblast-HSAS1 and HUVEC (Jennio Biotech Co. Ltd., Guangzhou, China) were sterilized under high temperature and pressure. The cells were suspended in the culture medium to obtain a cell density of 4 × 10^5^ cells mL^−1^, as counted by a hemocytometer. The cells were seeded on the biocomposite film with a density of 2 × 10^4^ cells in the wells of the tissue culture plates and maintained in culture medium supplemented with a 10% of fetal bovine serum and 10% of the pancreatic enzyme-EDTA in a 37°C incubator (SCO6WE, SHEL LAB) with 5% CO_2_. On 48 h, 96 h, and 144 h (or 2, 4, and 6 days) of in vitro culture, two cells were stained by 10 *μ*L calcein AM and 10 *μ*L polyimide for 15-20 min and then washed three times with PBS. The morphologies of the cells on the hydrogel surfaces were obtained by using a laser scanning confocal microscope (Leica TCS SP8, Germany).

## Data Availability

All data needed to evaluate the conclusions in the paper are present in the paper and/or the Supplementary Materials. Additional data related to this paper may be requested from the authors.

## Supplementary Materials

Supplementary Materials Further experimental characterization: SEM image and biocompatibility tests of degradable biocomposite film. Device fabrication, proton density simulation, and humidity distribution simulation. Mechanical properties of degradable biocomposite film-based flexible sensor. Humidity performances (e.g., sensitivity, response/recovery time, and stability) of degradable biocomposite film-based flexible sensor.
