# Supplementary material for: Biocompatible and Biodegradable Functional Polysaccharides for Flexible Humidity Sensors
Source: Research (Wash D C). 2020 Apr 9;2020:8716847. doi: 10.34133/2020/8716847 (PMC7171591; doi:10.34133/2020/8716847)
Supplement: Supplementary Materials — Further experimental characterization: SEM image and biocompatibility tests of degradable biocomposite film. Device fabrication, proton density simulation, and humidity distribution simulation. Mechanical properties of degradable biocomposite film-based flexible sensor. Humidity performances (e.g., sensitivity, response/recovery time, and stability) of degradable biocomposite film-based flexible sensor. [file 8716847.f1.doc]

Supplementary Materials

Biocompatible and Biodegradable Functional Polysaccharides for Flexible Humidity Sensors

Lili Wang^1^, Zheng Lou^2^*, Kang Wang^1^, Shufang Zhao^2^, Pengchao Yu^3^, Wei Wei^4^, Dongyi Wang^1^, Wei Han^3,5^, Kai Jiang^6^, Guozhen Shen^2^*

1 State Key Laboratory on Integrated Optoelectronics, College of Electronic Science and Engineering, Jilin University, Changchun 130012, PR China

2 State Key Laboratory for Superlattices and Microstructures, Institute of Semiconductors, Chinese Academy of Sciences, Beijing 100083, China

3 Sino-Russian International Joint Laboratory for Clean Energy and Energy Conversion Technology, College of Physics, Jilin University, Changchun 130012, P. R. China

4 Laboratory of Theoretical and Computational Chemistry, Institute of Theoretical Chemistry, Jilin University, Changchun, 130012, PR China

5 International Center of Future Science, Jilin University, Changchun 130012, P.R. China

6 Institute & Hospital of Hepatobiliary Surgery, Key Laboratory of Digital Hepatobiliary Surgery of Chinese PLA, Chinese PLA Medical School, Chinese PLA General Hospital, Beijing 100853, China

Corresponding author. E-mail: zlou@semi.ac.cn (Z. L), gzshen@semi.ac.cn (G. Z. S.)


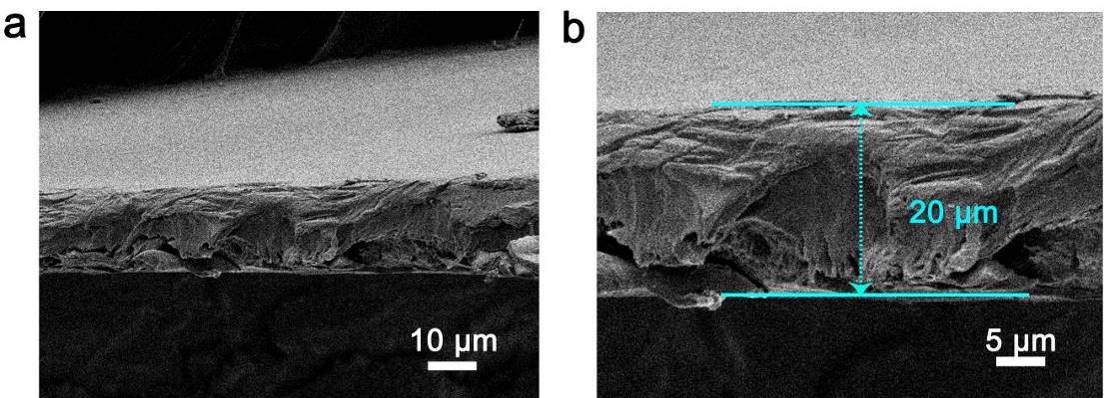


fig. S1. Degradable biocomposite film SEM characterization. SEM image of degradable biocomposite film with a thickness of approximately 20 μm: (A) high magnification and (B) low-magnification.


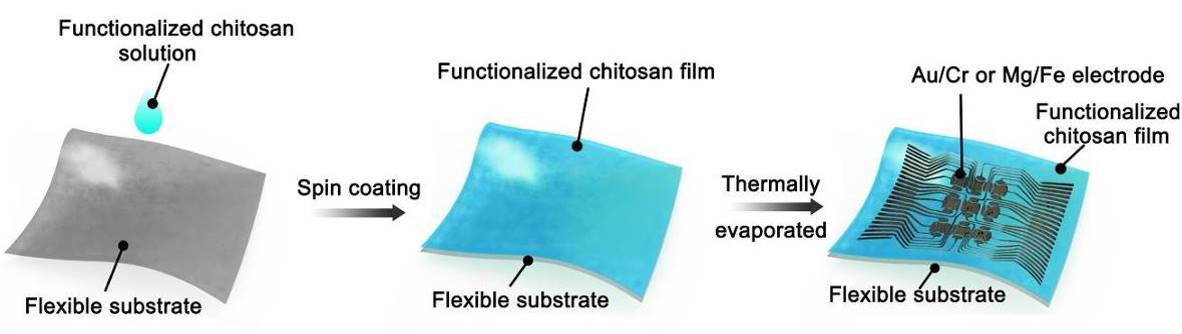


fig. S2. Device fabrication. Fabrication of degradable biocomposite film-based flexible humidity sensor.


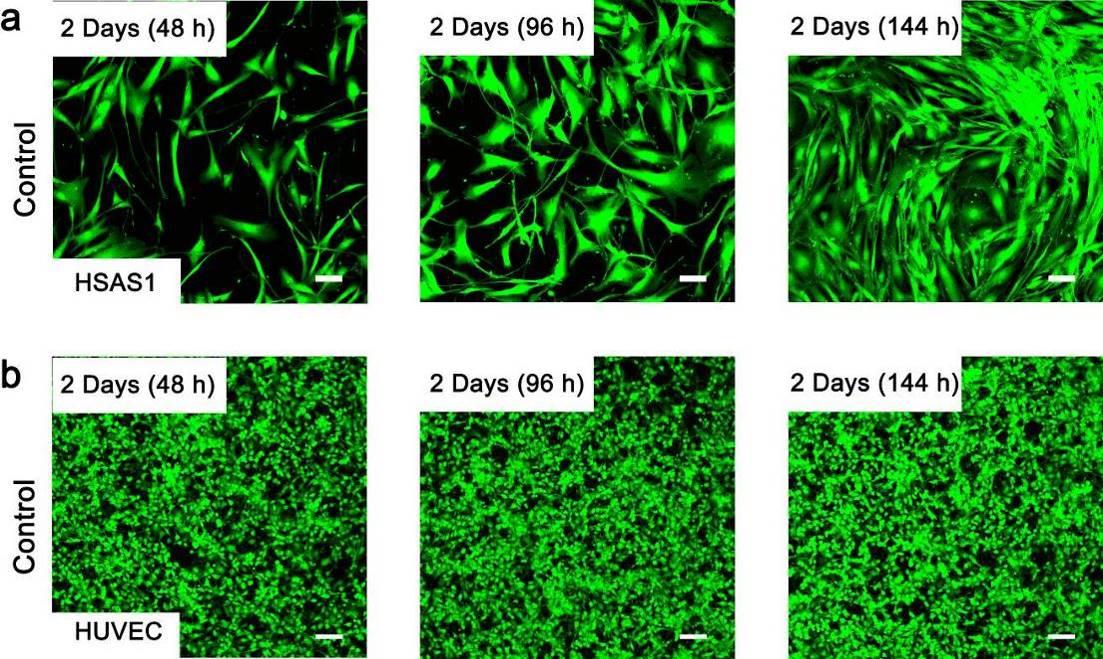


fig. S3. Biocompatibility tests. Confocal laser scanning microscopy images of (A) stained human skin fibroblasts-HSAS1 and (B) HUVEC that were cultured on blank film as control experiment. (Scale bar: 100 μm).


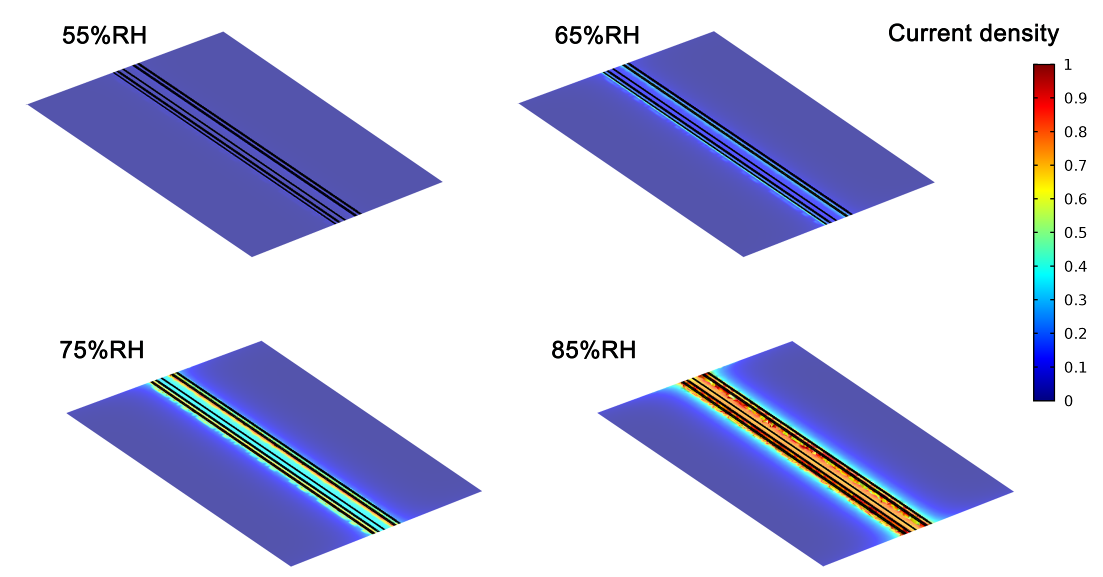


fig. S4. Proton density simulation. Simulated proton density distribution in degradable biocomposite film-based device under different humidity conditions.


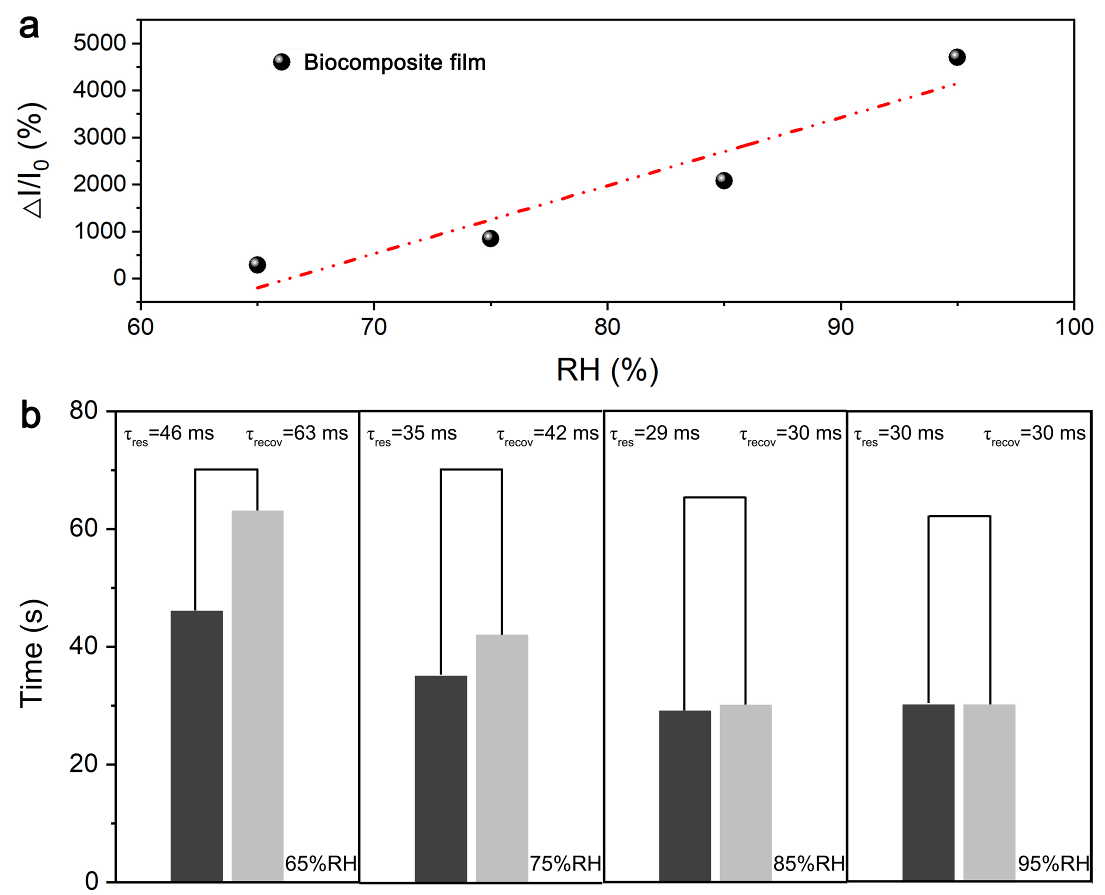


fig. S5. Humidity sensing performance. (A) Sensitivity of degradable biocomposite film-based flexible sensor to 65-95%RH. (B) Response time and recovery time of degradable biocomposite film-based flexible sensor to 65-95%RH.


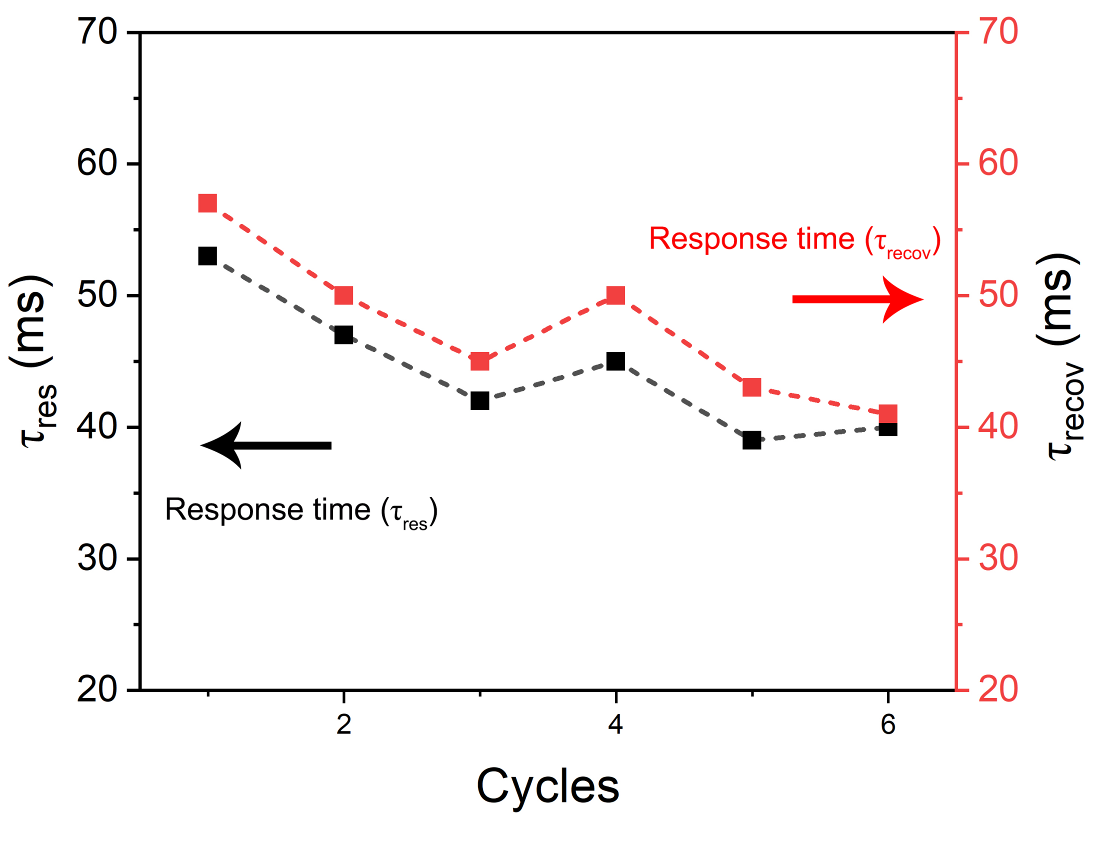


fig. S6. Response/recovery characterizations. Response time and recovery time of degradable biocomposite film-based flexible sensor for six cycles tests.


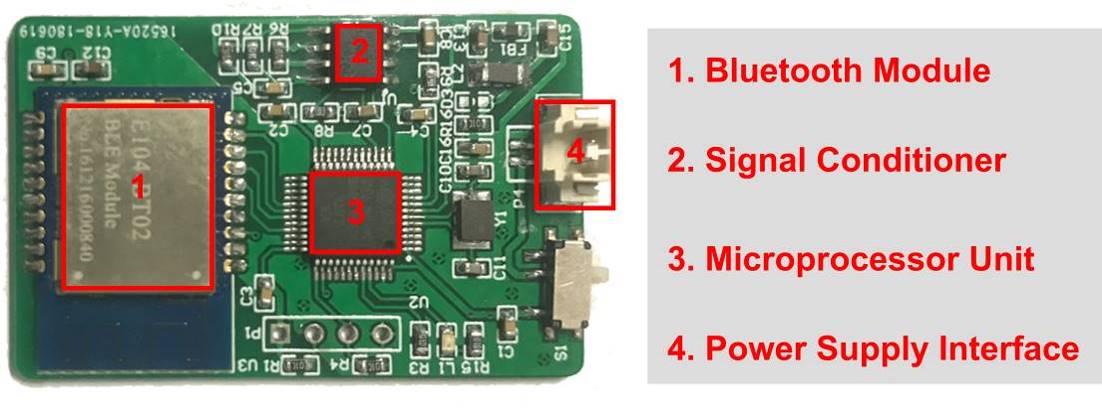


fig. S7. Structure of flexible humidity sensor. Picture and the main part of the printed circuit board.


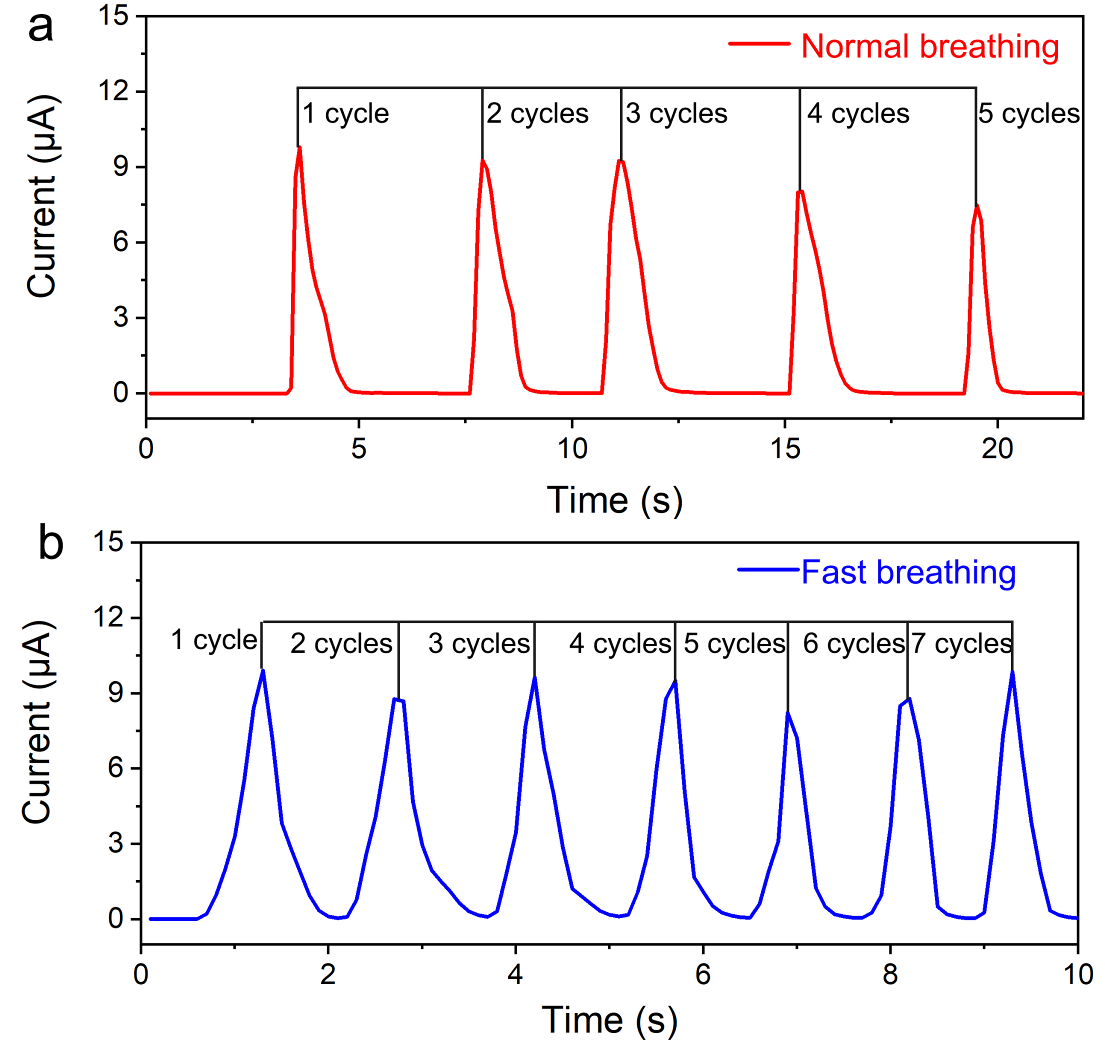


fig. S8. Respiration rate changes tests. Measured respiration rate changes in (A) normal breathing state and (B) fast breathing state.


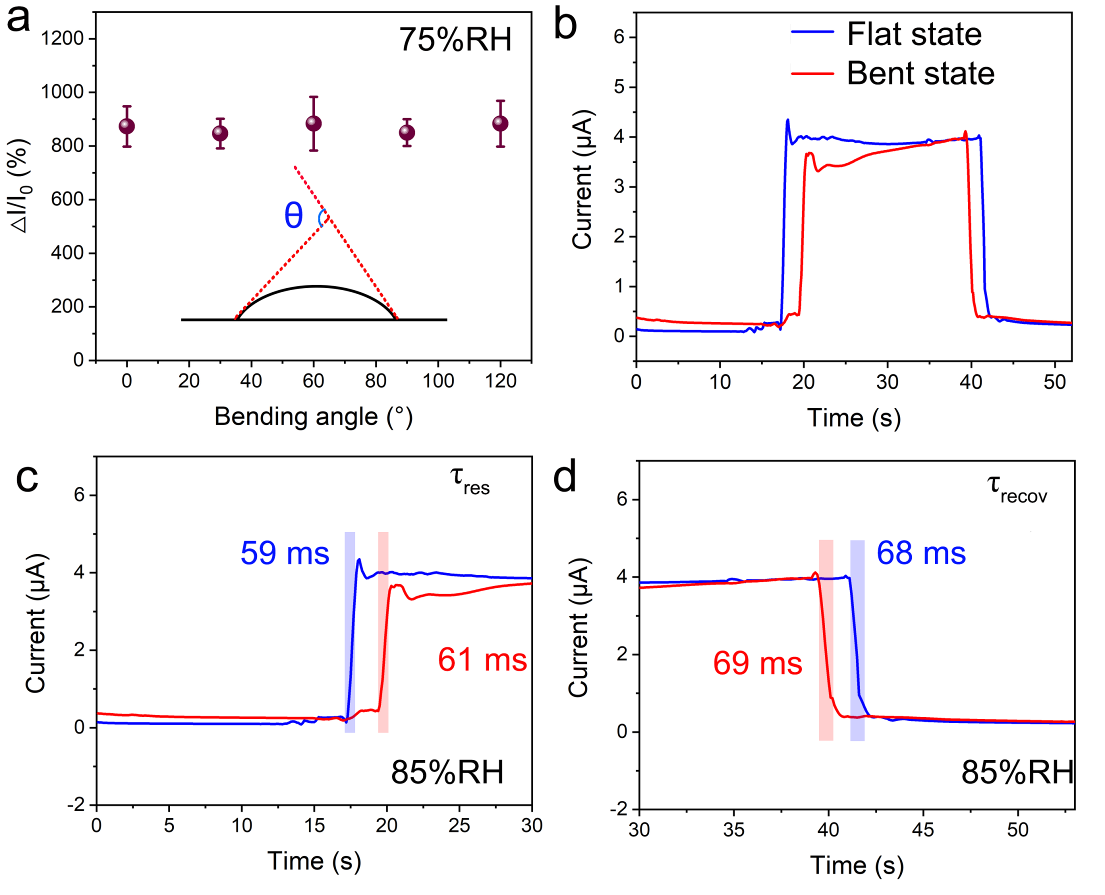


fig. S9. Mechanical properties. (A) Sensitivity of degradable biocomposite film-based flexible humidity sensors to 75%RH under different bending angles (n=3 measurements). (B) Dynamic single cycle sensing response of flexible sensor under flat and bent state. (C) Response time and (D) recovery time of flexible sensor to 85%RH.


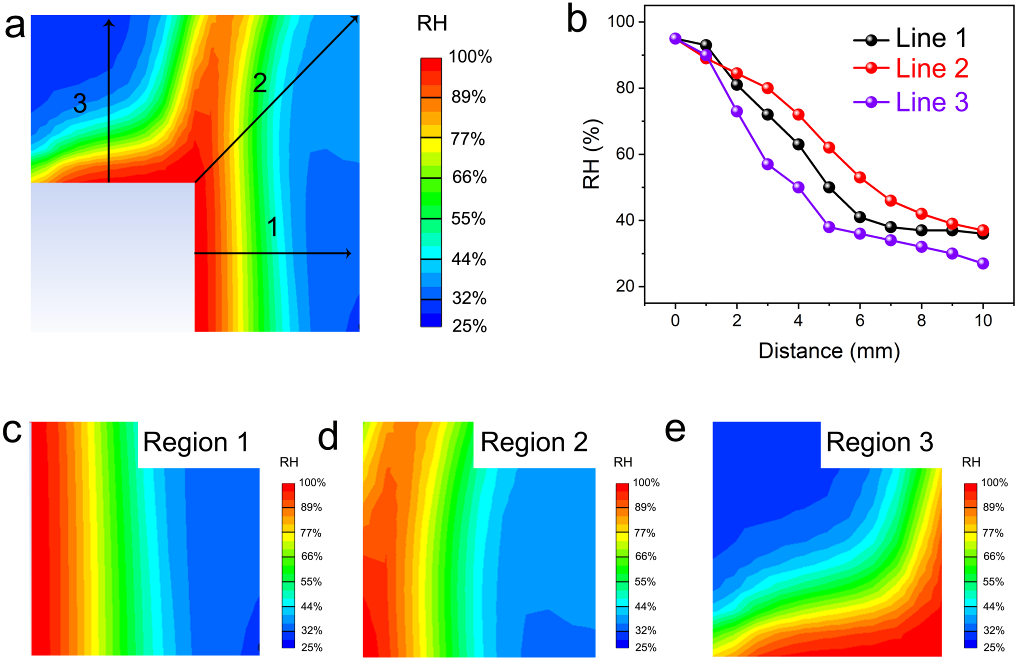


fig. S10. Humidity distribution simulation. (A) Cross-sectional FEM image shows humidity distribution with lateral color gradient around the finger position (10 mm). (B) Relationship curves of RH and distance (x) at position 1, 2 and 3 conducted by FEM analysis. Corresponding to humidity distribution with lateral color gradient around the position 1 (C), position 2 (D) and position 3 (E).


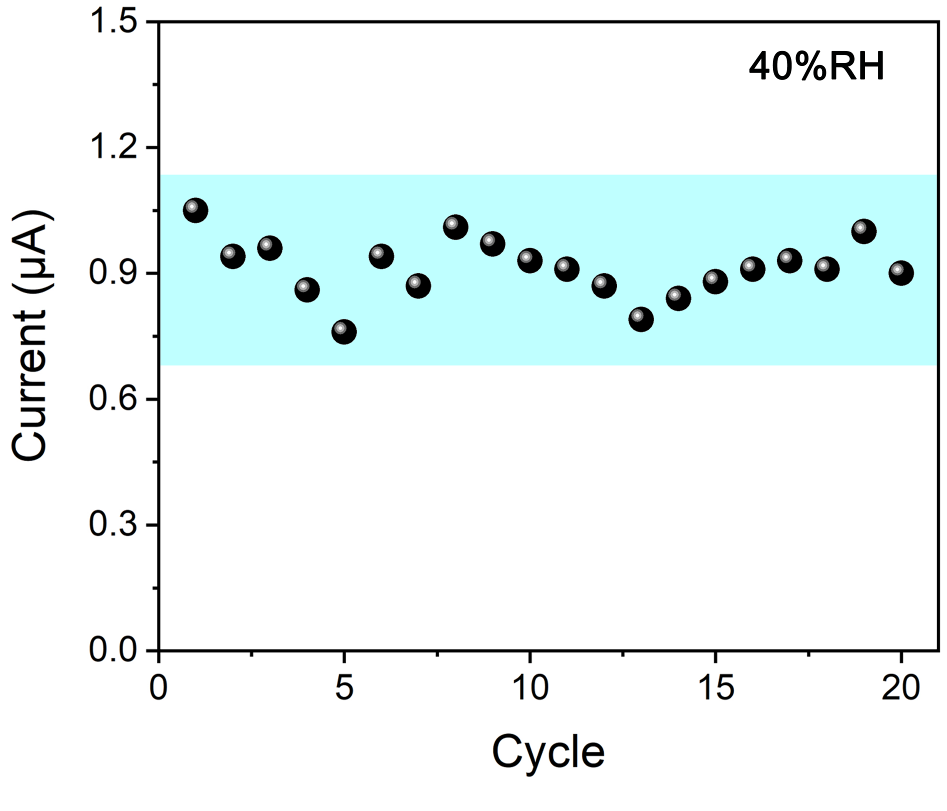


fig. S11. Stability of flexible sensor. The current response of flexible sensor to 40%RH at 20 cycles.


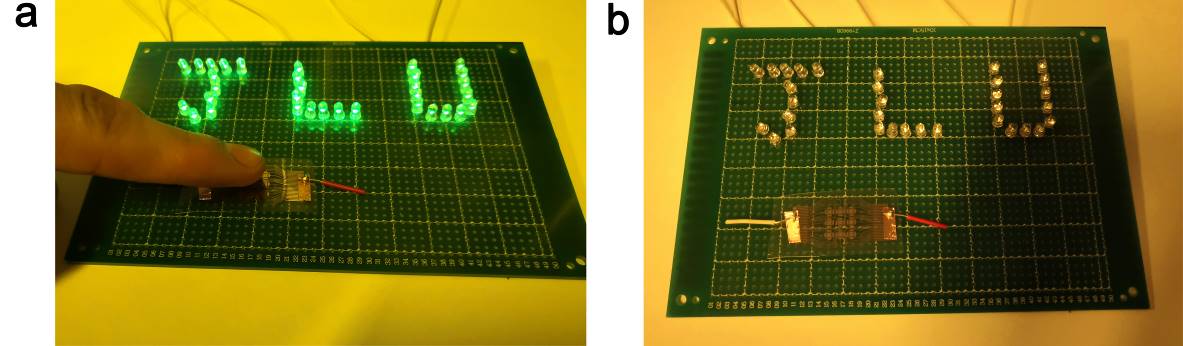


fig. S12. Application of the non-contact flexible sensor. Optical image of LED light brightness change of the non-contact flexible sensor with (A) and without (B) finger approached.


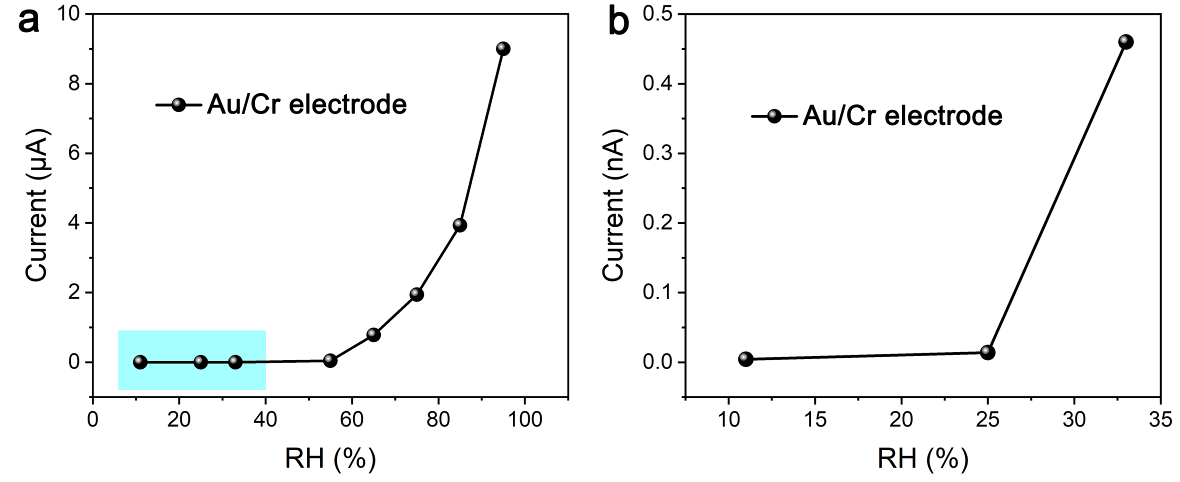


fig. S13. Sensing performance of flexible sensor with Au/Cr electrodes. The humidity sensing response of flexible sensor with Au/Cr electrodes under different RH levels: (A) 11%-95%RH and (B) 11%-33%RH.


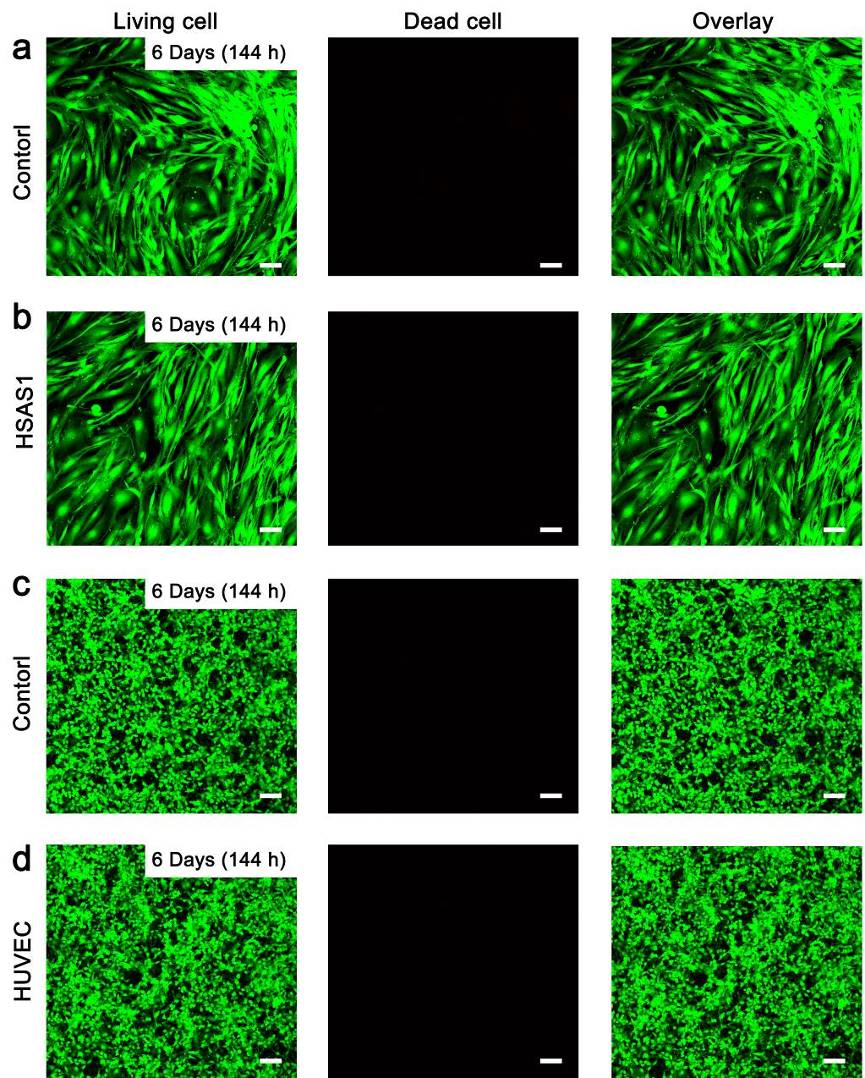


fig. S14. The cytotoxicity test. Confocal laser scanning microscopy images of (A, B) stained human skin fibroblasts-HSAS1 and (C, D) HUVEC that were cultured on functionalize polysaccharide film at 6 days (144 h). (Scale bar: 100 μm).

table S1 Comparison of sensing performance and biofunctionality of various humidity sensors

| Materials | RH/% | Sensitivity/△I/I_0_ (%) | T_res_/s | T_recov_/s | biofunctionality | Ref. |
| --- | --- | --- | --- | --- | --- | --- |
| Functionalized polysaccharides | 85 | 2500% | 0.29 | 0.30 | Flexibility  Biocompatibity  biodegradatily | This work |
| MoS_2_ | 35 | 10-10^-4^  (V_g_=-10-80V) | 10 | 60 | Flexibility | [1] |
| rGO | 4.3 | About 1% | 4 | 10 | -- | [2] |
| GO | 90 | -- | 0.30 | 0.30 | Flexibility | [3] |
| MoO_3_ | 40 | -- | 0.40 | 0.30 | Flexibility | [4] |
| SnS_2_/Zn_2_SnO_4_ | 97 | -- | 18 | 1 | -- | [5] |
| silica NP film | 84 | -- | 31.4 | 6.5 | Flexibility | [6] |

Supplementary References

[1] J. Zhao et al., Highly Sensitive MoS_2_ Humidity Sensors Array for Noncontact Sensation. *Adv. Mater.* 29, 1702076 (2017).

[2] X. W. Wang, Z. P. Xiong, Z. Liu, T. Zhang, Exfoliation at the Liquid/Air Interface to Assemble Reduced Graphene Oxide Ultrathin Films for a Flexible Noncontact Sensing Device. *Adv. Mater.* 27, 1370 (2015).

[3] S. Borini, R. White, D. Wei, M. Astley, S. Haque, E. Spigone, N. Harris, J. Kivioja, T. Ryhanen, Ultrafast Graphene Oxide Humidity Sensors. *ACS Nano* 7, 11166–11173 (2013).

[4] J. Yang, R. Shi, Z. Lou, R. Chai, K. Jiang, G. Z. Shen, Flexible smart non-contact control systems with ultrasensitive humidity sensors. *Small*, (2019)

[5] D. Z. Zhang, X. Zong, Z. Wu, Y. Zhang, Hierarchical Self-Assembled SnS_2_ Nanoflower/Zn_2_SnO_4_ Hollow Sphere Nanohybrid for Humidity Sensing Applications. *ACS Appl. Mater. Interfaces* 10, 32631-32639 (2018).

[6] S. Kano, M. Fujii, All-Painting Process to Produce Respiration Sensor Using Humidity Sensitive Nanoparticle Film and Graphite Trace. *ACS Sustainable Chem. Eng.* 6, 12217−12223 (2018).
